# A Rare Case of Rapidly Progressive Haemophilus Parainfluenzae Endocarditis With Cerebral Emboli

**DOI:** 10.7759/cureus.85347

**Published:** 2025-06-04

**Authors:** Zarifa Orta, Murat Hakan Kir, Selva Ala Selek, Aysun Benli, Serap Simsek Yavuz

**Affiliations:** 1 Infectious Diseases and Clinical Microbiology, Tekirdag Dr. İsmail Fehmi Cumalıoglu City Hospital, Tekirdag, TUR; 2 Infectious Diseases, Istanbul University School of Medicine, Istanbul, TUR; 3 Infectious Diseases, Bagcılar Hospital, Istanbul, TUR

**Keywords:** endocarditis, haemophilus parainfluenzae, mitral valve prolapse, septic embolism, valvular vegetation

## Abstract

*Haemophilus parainfluenzae*, a member of the HACEK group of bacteria, is a rare causative agent of infective endocarditis IE. Typically presenting with a subacute course, *Haemophilus parainfluenzae* infective endocarditis can, in rare cases, progress acutely, leading to severe complications. This case report highlights a rapid onset of infective endocarditis caused by *Haemophilus parainfluenzae*, a pathogen not commonly associated with aggressive disease. The infection, accompanied by septic emboli and cardiac involvement, underscores the need for early diagnosis, prompt treatment, and vigilant follow-up. Our patient presented with an acute clinical picture, featuring fever, confusion, and rapid neurological deterioration, necessitating early intervention and targeted therapy. This rapid progression, compounded by cerebral and cardiac complications, highlights the unusual nature of this infection and the challenges it presents in clinical management. The presence of neurological symptoms in a young patient with no history of chronic illness should raise suspicion for IE, especially when accompanied by fever and embolic phenomena. Early empirical treatment is crucial, as it can prevent progression to more severe manifestations. This report also emphasizes the importance of thorough cardiac evaluation in young patients with unexplained fever and neurological symptoms. Structural heart conditions, such as mitral valve prolapse, may predispose individuals to IE, and early detection of such conditions can guide diagnosis and treatment. In our case, the mitral valve involvement was not initially considered, delaying the suspicion of IE. However, once the diagnosis was confirmed, targeted therapy led to a positive outcome.

*Haemophilus parainfluenzae* IE, though rare, can present acutely with severe complications, including septic emboli and cardiac involvement. This report emphasizes the need for clinicians to maintain a high index of suspicion for infective endocarditis in young patients with persistent fever and neurological symptoms, particularly when embolic manifestations are present. Early recognition, aggressive treatment, and comprehensive follow-up are essential for managing this rare but serious condition. Additionally, this report reinforces the importance of a thorough cardiac evaluation in the differential diagnosis of young patients presenting with fever and embolic events.

## Introduction

Infective endocarditis (IE) is a rare but serious infection of the heart valves, typically caused by common pathogens such as *Staphylococcus aureus (S. aureus)*, *Streptococcus* species, and *Enterococcus* species. However, less frequent etiologies, including the HACEK group of bacteria - *Haemophilus* species, *Aggregatibacter* species, *Cardiobacterium hominis*, *Eikenella corrodens*, and *Kingella kingae *- account for approximately 1-3% of all IE cases [1]. Among these, *Haemophilus parainfluenzae (H. parainfluenzae)* is a fastidious, Gram-negative coccobacillus that forms part of the normal flora of the human upper respiratory tract [2].​ Historically, *H. parainfluenzae* IE has been associated with a subacute course, often affecting patients with underlying valvular abnormalities. However, recent reports have highlighted more aggressive presentations, including acute onset, larger vegetations, and increased rates of septic emboli, particularly to the central nervous system [3]. The clinical management of *H. parainfluenzae* IE typically involves prolonged antibiotic therapy with third-generation cephalosporins, such as ceftriaxone, and may require surgical intervention in cases of heart failure or persistent infection. Despite its rarity, the evolving clinical patterns and potential for severe complications underscore the importance of early recognition and appropriate treatment of this condition. Diagnosis is often delayed due to its slow growth in standard blood cultures. We present a case of acute *H. parainfluenzae* endocarditis complicated by meningoencephalitis and cerebral embolism.

## Case presentation

A 23-year-old female presented with a four-day history of fever and confusion. She had previously sought medical attention for fever and sore throat, receiving amoxicillin-clavulanic acid. A day before admission, her fever had persisted despite paracetamol, accompanied by severe headache and confusion. She had no chronic illnesses, recent animal exposure, or consumption of unpasteurized dairy, but had a history of freshwater scuba diving two months earlier. On examination, she was febrile (38.5 °C), tachycardic (120 bpm), and hypotensive (90/60 mmHg). She exhibited neck stiffness but no other focal neurological deficits. Initial laboratory results revealed leukocytosis, thrombocytopenia, and elevated inflammatory markers (Table 1).

**Table 1:** Laboratory parameters High leukocyte, CRP, and levels CRP: C-reactive protein; HBsAg: hepatitis B surface antigen; HCV: hepatitis C virus; HIV: human immunodeficiency virus; PCT: procalcitonin

Parameter	Two days before admission	At admission	Reference range
Leukocyte (mm³)	8,600	13,520	4,000-11,000
Hemoglobin (g/dl)	11.8	11.5	12.0-16.0
Platelet (mm³)	189,000	105,000	150,000-400,000
Neutrophil (mm³)	7,760	12,440	1,800-7,700
Lymphocyte (mm³)	770	710	1,000-4,800
Creatinine (mg/dl)	0.69	0.63	0.5-1.1
CRP (mg/l)	42	190	<5
PCT (ng/ml)	-	10.8	<0.5
Fibrinogen (mg/dl)	-	568	200-400
Anti-HIV	-	Negative	Negative
HBsAg	-	Negative	Negative
Anti-HCV	-	Negative	Negative

**Figure 1:**
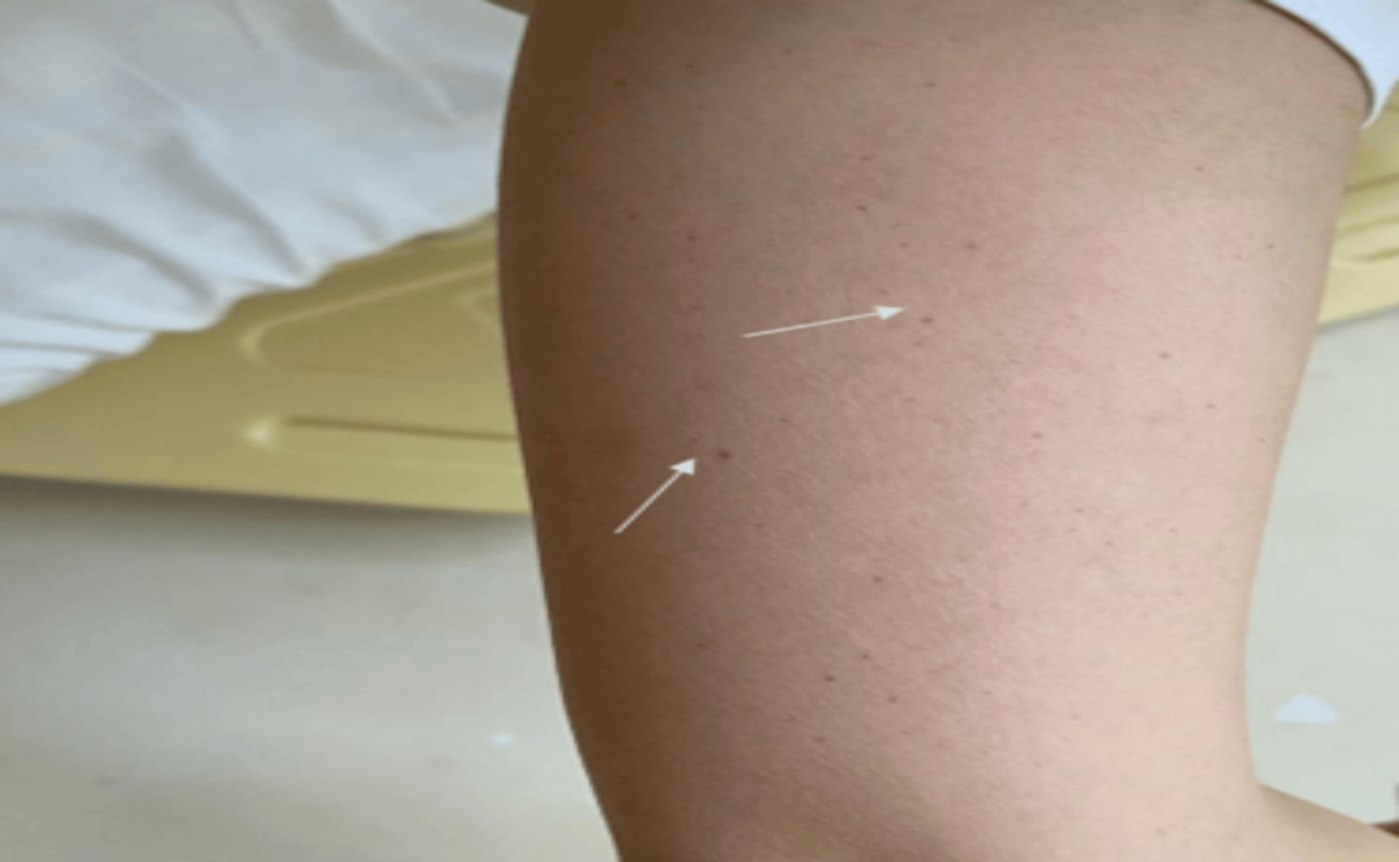
Petechial hemorrhages on the medial aspect of the right upper extremity (arrows)

Lumbar puncture showed pleocytosis (425 PMNs/mm³, 80 lymphocytes/mm³), with elevated protein (0.52 g/L) and lactate (2.5 mmol/L). A Gram stain of the cerebrospinal fluid (CSF) showed no microorganisms. Empirical ceftriaxone (2x2 g IV) was initiated for presumed bacterial meningoencephalitis. By day two, her consciousness improved, but fever and thrombocytopenia persisted. Petechial hemorrhages were noted on the right upper extremity (Figure 1) and back.

**Figure 2:**
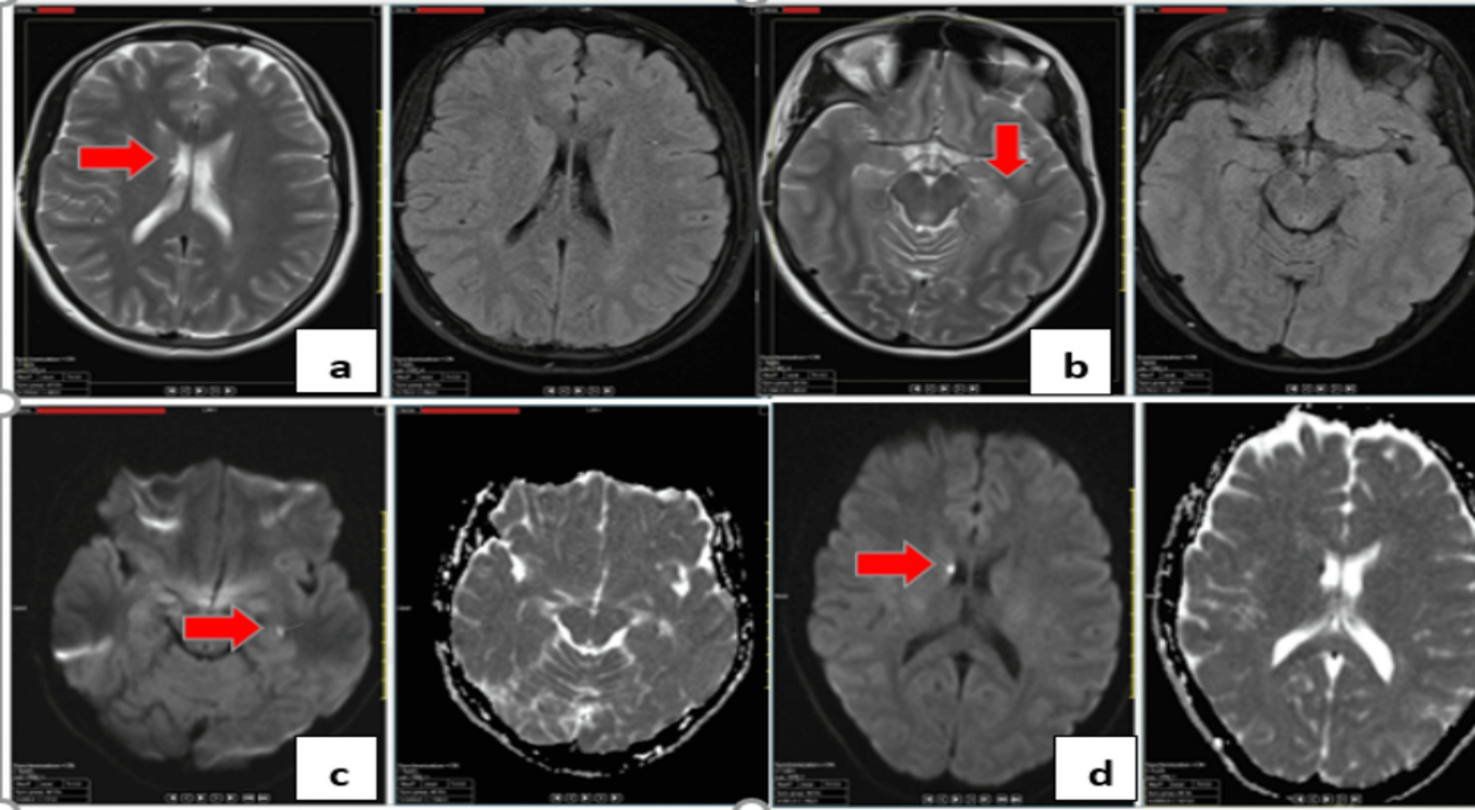
Cranial MRI a) T2A/FLAIR signal increase on the left ependymal surface and periventricular ependymal surfaces; b) edema in the occipital region; c) diffusion restriction in the left hippocampus and d) right caudate nucleus, and medial temporal lobe FLAIR: fluid-attenuated inversion recovery; MRI: magnetic resonance imaging

**Figure 3:**
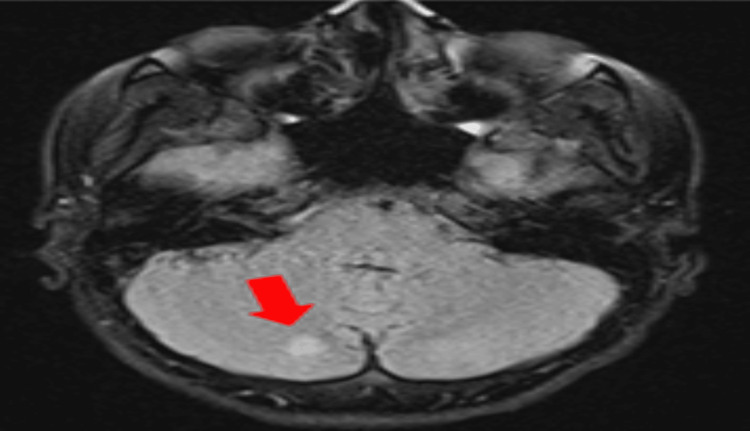
Control cranial MRI A new embolus was identified in the inferior region of the right cerebellar hemisphere MRI: magnetic resonance imaging

**Figure 4:**
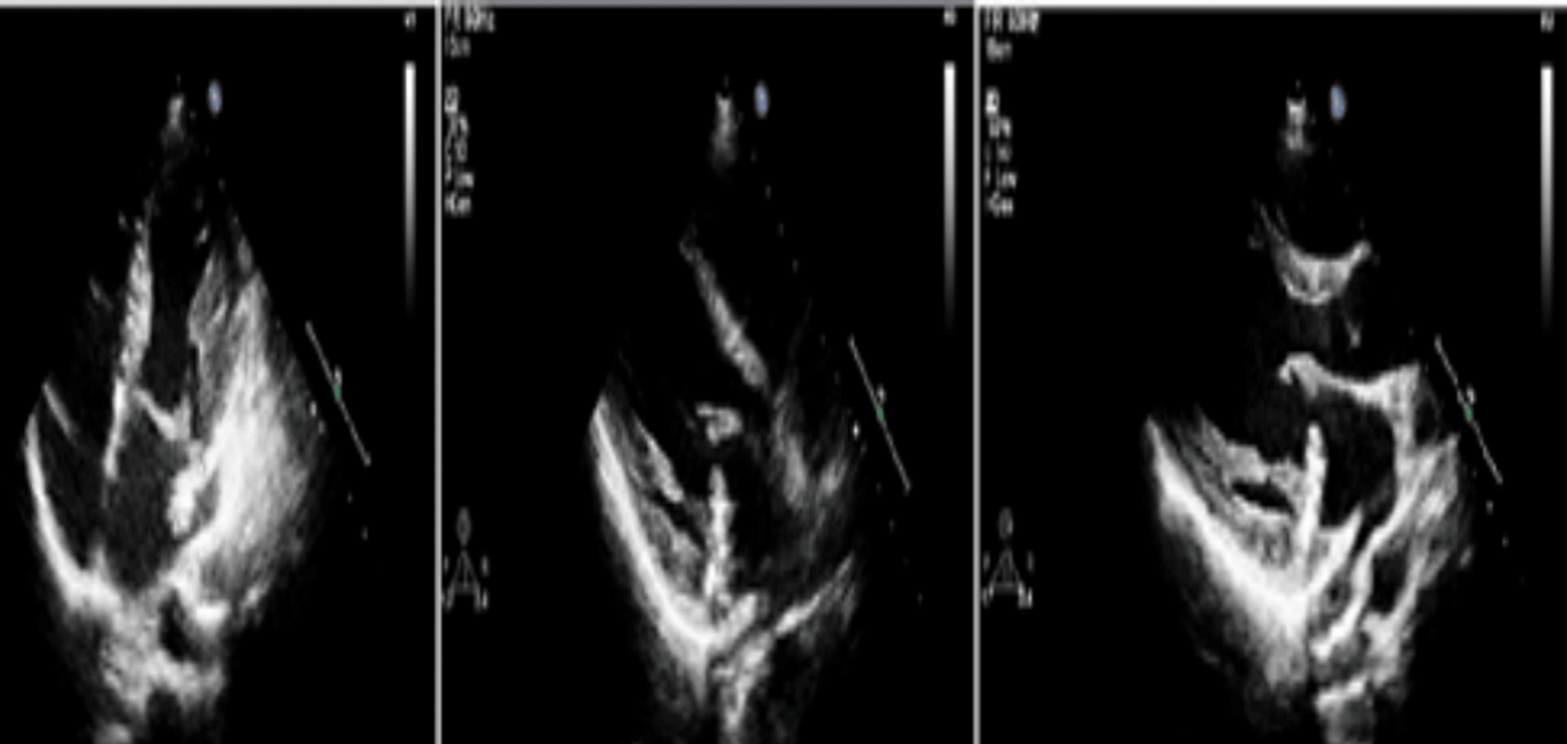
TTE examination The posterior cusp of the mitral valve was redundant, mobile, hypermobile, measuring 15-16 mm in length and 6-7 mm in width, prolapsing without restriction. A mass-like image compatible with vegetation was observed on its atrial surface. Systolic annular dysfunction of approximately 12 mm was noted in the mitral annulus lateral region TTE: transthoracic echocardiography

Meningococcemia and gonococcemia were considered, prompting the addition of meropenem and vancomycin. Cranial MRI revealed T2a/flair signal abnormalities (Figure 2). Blood cultures yielded Gram-negative coccobacilli, later identified as *H. parainfluenzae* via matrix-assisted laser desorption/ionization time-of-flight (MALDI-TOF) mass spectrometry. On day four, she developed a dry cough, and chest imaging revealed right lower lobe pleural effusion. Elevated pro-BNP suggested cardiac involvement. A new systolic murmur prompted a transthoracic echocardiography (TTE), which revealed a mobile, hyperechoic vegetation (11 mm) on the posterior mitral leaflet and mitral valve prolapse. Transesophageal echocardiography (TEE) confirmed these findings. The diagnosis of mitral valve endocarditis due to *H. parainfluenzae* was established. Despite targeted ceftriaxone therapy, her fever persisted, and a follow-up MRI showed new embolic lesions in the cerebellum (Figure 3). On day 13, repeat TTE showed vegetation enlargement (15-16 mm) (Figure 4).

Cardiovascular surgery recommended valve replacement, and the patient underwent mitral valve replacement with a mechanical prosthesis on day 16. Postoperatively, fever resolved, and inflammatory markers normalized. After six weeks of targeted therapy, she was discharged in stable condition. The clinical timeline is detailed in Table 2.

**Table 2:** Clinical timeline BNP: brain natriuretic peptide; CRP: C-reactive protein; CT: (computed tomography); CVS: cardiovascular surgery; MRI: magnetic resonance imaging; PCT: procalcitonin; PET: positron emission tomography; TEE: transesophageal echocardiogram; TTE: transthoracic echocardiogram

Day	Event/observation
0	Fever, confusion, and sore throat. Amoxicillin-clavulanic acid started
2	Consciousness improved, but remained drowsy, and fever persisted. CRP increased to 220 mg/l, and PCT rose to 25 ng/ml. Platelet count dropped to 58,000/mm³, D-dimer was 2208 µg/l. A peripheral blood smear revealed rare schistocytes, but disseminated intravascular coagulation was not suspected. Petechial hemorrhages noted. Contrast-enhanced cranial MRI showed T2a/flair signal increase. Blood culture from her initial visit yielded a positive signal, and Gram-negative coccobacilli were seen on Gram staining
4	Developed dry cough, auscultation revealed decreased breath sounds at the right lung base. Thorax CT revealed a 3 cm right lower lobe and minimal left pleural effusion. With a pro-BNP 2150 pg/ml, cardiac overload was suspected. Blood culture specimen on chocolate agar yielded growth, while standard blood agar did not, with Gram-negative coccobacilli observed on staining from a second culture bottle
6A	A 2/6 systolic murmur was detected over the mitral focus; a TTE was performed. The TTE revealed mitral valve prolapse and a mobile, hyperechoic, irregularly surfaced mass attached to the atrial surface of the posterior mitral leaflet, measuring up to 11 mm, suggestive of vegetation. *H. parainfluenzae* identified, treatment adjusted to ceftriaxone.
10	Suspicious for septic emboli secondary to infective endocarditis, the patient underwent abdominal ultrasound and PET/CT, but no emboli were detected. The patient was referred to CVS. Consultation sought for potential surgical intervention, but continuation of medical treatment was recommended. Due to persistent fever, a contrast-enhanced cranial MRI was performed to evaluate for septic emboli in the brain, and a non-enhancing new embolus was identified in the inferior region of the right cerebellar hemisphere
13	Follow-up TEE showed an increase in vegetation size. Surgery was recommended
16	Mitral valve excised and replaced with a mechanical valve. Patient started improving, treatment completed

## Discussion

IE is a life-threatening condition characterized by infection of the heart valves. The most common causative organisms include* S. aureus*, viridans group streptococci, and enterococci; however, *H. parainfluenzae*, a member of the HACEK group, remains a rare yet notable pathogen [1]. HACEK organisms account for 1-3% of IE cases, with *H. parainfluenzae* being an infrequent cause. A 20-year review identified only 39 adult cases of *H. parainfluenzae* IE [2]. Unlike the typical subacute course, our case presented acutely with fever and neurological symptoms, highlighting the potential for aggressive disease progression. Similar acute presentations have been described in rare cases, where *H. parainfluenzae *caused large vegetations and embolic events, mimicking more virulent pathogens such as *S. aureus* [3,4]. Septic emboli are common in *S. aureus*, *Candida* spp., and HACEK-related IE, with approximately 70% of embolic complications involving the central nervous system [2,5].

Although headache is an uncommon initial symptom of IE, it may indicate cerebral embolism [6]. Neurological manifestations can be among the first signs of IE and are associated with increased morbidity and mortality [7]. Early empirical treatment likely prevented a more severe course in our patient, underscoring the importance of timely intervention. Data in the literature show that neurological complications in IE are associated with a poorer prognosis, particularly when diagnosis is delayed [8]. Although the slow growth of *H. parainfluenzae* in cases presented in the literature delayed the diagnosis and the treatment, our patient was promptly diagnosed and treated, based on close follow-up of symptoms and clinical suspicion. In addition, isolating the causative agent supports the importance of blood and urine cultures before starting antibiotic treatment [9]. Structural heart valve diseases such as mitral valve prolapse (MVP) have been associated with a four-to-eight-fold increased risk of IE, especially when accompanied by mitral regurgitation [10]. Since the history of MVP was not known at the time of presentation in our patient, IE was not initially considered in the differential diagnosis. This underscores the need for thorough cardiac evaluation in young patients presenting with fever and embolic phenomena.

## Conclusions

This report discussed a rare case involving the acute onset of *H.* *parainfluenzae* IE, presenting with neurological symptoms and rapid disease progression, unlike the typical subacute course. Clinicians should maintain a high index of suspicion for IE in young patients with persistent fever and neurological symptoms, particularly when accompanied by embolic manifestations. This report also highlights the rare but serious nature of *H. parainfluenzae* endocarditis, emphasizing the need for early recognition, aggressive treatment, and follow-up imaging in detecting complications. Such cases necessitate further investigations, and these efforts are of paramount importance for the diagnostic process.
